# Supplementary material for: Integrated analysis of the aqueous humor microbiome and lens capsule transcriptome in high myopia cataract: a pilot study
Source: Front Med (Lausanne). 2026 Jun 16;13:1845205. doi: 10.3389/fmed.2026.1845205 (PMC13314463; doi:10.3389/fmed.2026.1845205)
Supplement: Supplementary file 3 [file Data_Sheet_1.zip › 3.Beta_Diversity/PCoA/C372089/PCoA_euclidean_2D_nolabel.pdf]

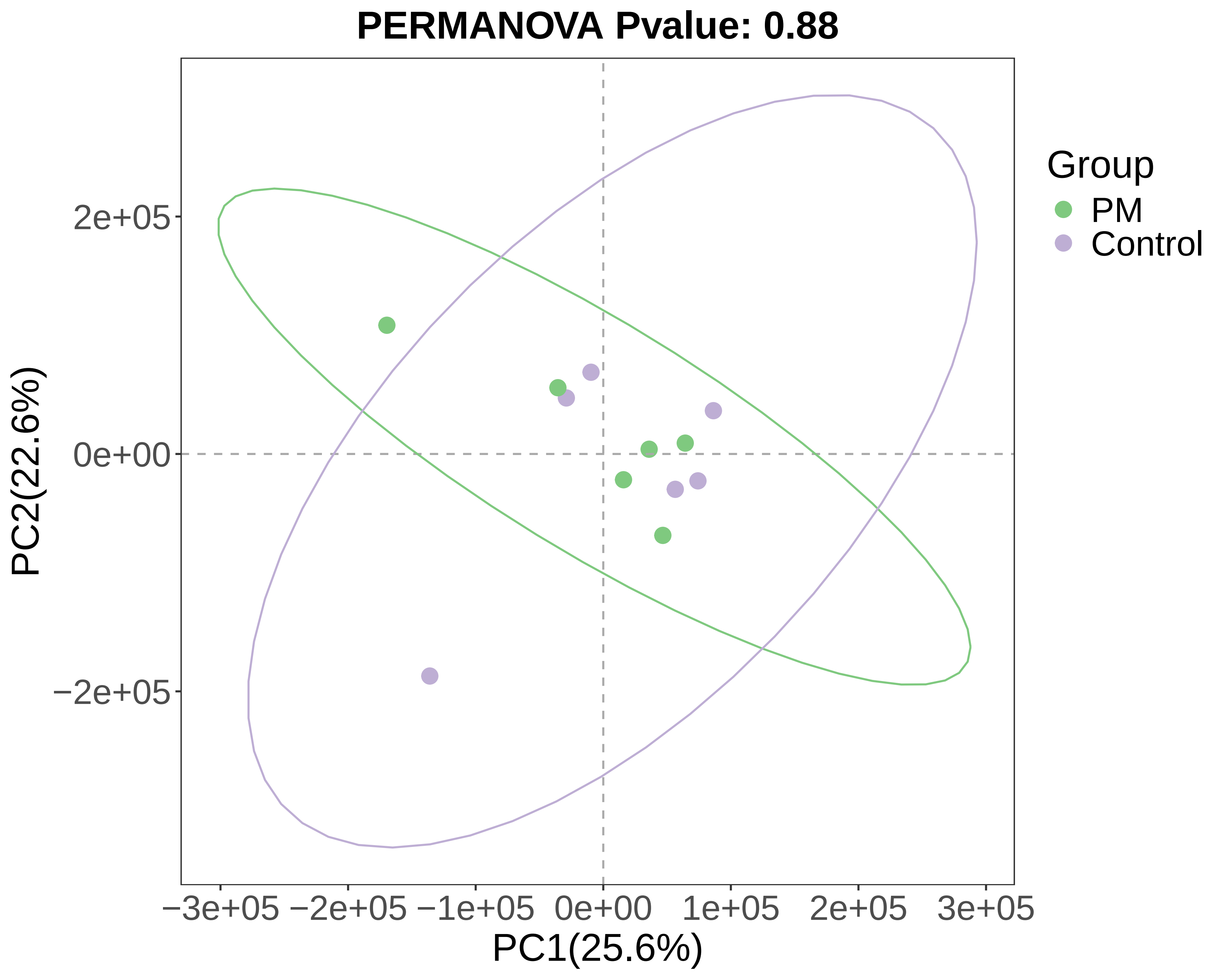
PERMANOVA Pvalue: 0.88
PC2(22.6%)
PC1(25.6%)
2e+05
0e+00
−2e+05
−3e+05
−2e+05
−1e+05
0e+00
1e+05
2e+05
3e+05
Group
PM
Control
